# Effect of porous structure of coal on propylene adsorption from gas mixtures

**DOI:** 10.1038/s41598-020-67472-x

**Published:** 2020-07-09

**Authors:** Karolina Wojtacha-Rychter, Natalia Howaniec, Adam Smoliński

**Affiliations:** 1 Department of Mining Aerology, Central Mining Institute, Pl. Gwarków 1, 40-166 Katowice, Poland; 2 Department of Energy Saving and Air Protection, Central Mining Institute, Pl. Gwarków 1, 40-166 Katowice, Poland; 3 Central Mining Institute, Pl. Gwarków 1, 40-166 Katowice, Poland

**Keywords:** Environmental sciences, Natural hazards, Chemistry, Engineering

## Abstract

This paper addresses the issue of the sorption process on coal concerning propylene released from the source of coal heating in the deposit. In this study, the interaction between Polish coals and propylene molecules, as well as three other hydrocarbons (ethylene, ethane, and propane) with the application of a fixed-bed column, was investigated. The experimental results show that propylene adsorption was measurable under the experimental conditions. The differences in the amount of adsorbed propylene were predominately caused by various gas diffusion rates within the pore network associated with the molecular sieving effect. According to the experimental results, the influence of mesopores on propylene adsorption was significantly stronger than the share of micropores of the explored coals. The column tests demonstrated that the largest amount of propylene was adsorbed by coal with the highest value of pore diameter (6.48 nm) determined by nitrogen adsorption at 77 K. Under the experimental conditions, the influence of other hydrocarbons and a surface area on the quantity of the adsorbed gas was unnoticeable. This study provides an understanding of the behavior of some of the fire gases during the flow of the mixture through a heterogeneous structure of coal in the mine environment. The sorption of gases from multi-component mixture released during the self-heating of coal on carbon materials such as bituminous and lignite coals is poorly understood which provides the rationale for the topic of this work.

## Introduction

An endogenous fire is a very complex process occurring in the coal deposit conducive to heat accumulation and in zones hard to reach for people, which makes it difficult to detect the fire at an early in its development. The hazard of an endogenous fire arises due to coal self-oxidation process^1–3^. As a consequence of the contact with molecular oxygen from the air, which is entered the mine through the ventilation systems, coal undergoes low-temperature oxidation^4–6^. In the initial stage, oxidation takes place on the surface of the macropores and near the outer surface of the grains and fractures^7^. In the next phase, the oxygen molecules diffuse through increasingly narrower micropores into the inner surface of the pores where they undergo chemisorption on active surface centers. On account of the exothermic coal oxidation reaction, heat is released. If the heat emitted exceeds heat dissipated, the temperature within the coal mass and the oxidation reaction rate start to rise. At the same time, the unstable oxygen complexes are decomposed to produce the main gaseous compounds such as carbon dioxide, carbon monoxide and water vapor^8,9^. In recent years, the application of the Gas Chromatography – Flame Ionization Detector (GC-FID) allowed also to detect certain amounts (at the level of parts per million) of saturated (ethane and propane) and unsaturated (ethylene, propylene and acetylene) hydrocarbons in mine atmosphere occurring with the increase in coal temperature^10,11^. An endogenous fire is a process that can spread uncontrollably. The detection of this process in its initial development phase allows for the safe evacuation of employees, as well as for an effective control of the heating process and liquidation of the fire, often without stopping the extraction of coal^12,13^. One of the most common methods of identifying fire hazards in the mining sector is the method on basis of monitoring the composition of mine atmosphere in the air currents flowing in and out of the controlled area as well as at goafs and insulation dams^14,15^. Next, the results of the analyses are compared with the numerical model of fire gases emissions obtained under laboratory coal oxidation. However, the composition of the mixture released from the source of coal self-ignition may change because of the sorption phenomenon.

Coal is a natural sedimentary raw material with enhanced sorption properties. Both microscopic studies and the measurements of carbon dioxide or nitrogen sorption isotherms have confirmed that coal has a very extensive pore system with the share of very narrow pores (below 2 nm), mesopores (from 2 to 50 nm) and macropores (above 50 nm)^16,17^. The porous structure of coal determines its sorption capacity^18,19^. The larger amounts of gases are mainly adsorbed in the micropores or narrow mesopores due to the enormous inner surface area and a large number of active sites accessible for gas adsorption^20,21^. In contrast, large mesopores and macropores play an essential role in adsorbate transport^22–24^. A different degree of organic matter decomposition (aerobic or anaerobic) influenced the formation of various petrographic groups in the biochemical stage. It was observed that the share of individual pores in the pore space of coal also depends on the petrographic composition^22,25,26^. Individual groups of macerals are characterized by different porosity and pore structure. Studies have shown that inertinite macerals are more porous than the vitrinite macerals, but they are characterized by a lower share of micropores. The content of macerals in parent coals was also reported to be affecting the porous structure properties of thermally processed coals under pressurized conditions^27,28^. After the biochemical phase of material decomposition, further transformation occurs under the influence of geochemical factors such as pressure and temperature. At this time, the carbon content is steadily increasing, while oxygen, hydrogen and volatile matter content decreases. With the increase in carbon content, the share of micropores increases at the expense of mesopores and macropores. In high rank coals, porosity is primarily determined by the presence of micropores^29^.

In a number of published research papers^30–33^, the adsorption of gases most frequently used in fire risk assessment was discussed. In most of the studies the adsorption of a single gas on coal or the mixture of carbon dioxide and methane as an adsorbant was measured, and only in the context of methane recovery. However, under real conditions, the gases emitted from the point of self-ignition of coal constitute a multi-component gaseous mixture moving through the porous structure of coal. In study by Dudzińska^34,35^ the measurements were performed on degassed coal samples with the use of pure gases as adsorbants. A comparative analysis of the amounts of adsorbed gases indicated that propylene is sorbed in a larger amount than saturated hydrocarbons, carbon monoxide and hydrogen. The adsorption of propylene is on a comparable level with ethylene adsorption, while it is lower than acetylene. It was concluded that the higher preferential sorption of propylene, ethylene and acetylene over ethane and propane saturated hydrocarbons is the result of the interactions involving the π electrons of the multiple bond and the active centers present on the surface of coal. The authors of the works^11,36–38^ analyzed the coal sorption properties with the application of a fixed-bed column method and the multicomponent gaseous mixtures as adsorbant. The results showed a selective adsorption of mixture components. Additionally, the findings presented in these studies demonstrated that propylene is the gas which is the most sensitive to concentration changes in samples taken under real conditions. This gas reached the saturation state in the longest time and was sorbed in a larger amount in comparison to the remaining gases monitored. The higher reactivity of propylene is related to the strength of C–H bonds and the stability of the carbocation^38^. In these works, the porous structure parameters of coal samples were determined only on the basis of the nitrogen isotherm. Thus, the influence of pore size distribution below 2 nm on the amount of propylene adsorbed was not accurately analyzed, due to the limitation of the nitrogen adsorption method. Generally, in most of the above cited publications, it was also concluded that coal with high porosity, a low degree of metamorphism, a high value of specific surface area and the volume of pores determined by micropores and narrow mesopores, as well as high oxygen content sorbed the largest amounts of hydrocarbons.

In the literature, the studies on propylene adsorption and its behavior in porous materials are mainly performed in the context of the development of an alternative olefin separation technology in the petrochemical industry^39–41^. The current standard practice for the separation is a cryogenic distillation which is an energy intensive process^40^. A very promising solution is a separation technique based on the different diffusion rates of gases in the pore network or on the difference in the strength of the interactions between gas molecules in the mixture and the adsorbent surface^41^.

In this paper, the impact of the porous structure parameters of selected Polish coals on propylene sorption using a fixed-bed column was analyzed. The porous structure characterization was performed on the basis of nitrogen sorption isotherm at liquid nitrogen temperature and carbon dioxide sorption isotherm at 273 K with the application of the Autosorb iQ analyzer (Quantachrome Instruments, USA). It allowed to explore the impact of pore size distribution also at a range of narrow microporosity (from 0.45 to 1.45 nm) on the quantity of propylene adsorbed on coal, which has not been done in previous works^42,43^. The effects of the pore size distribution, the pore volume, the average pore diameters as well as the specific surface area of selected adsorbents on their sorption properties towards selected adsorbates were discussed in the aspect of the correct assessment of the fire hazard status in underground mines. There are very few published studies providing data on the effect of propylene flow in the coal pore network and its adsorption. The porous structure of various materials i.e. zeolites, active carbons or natural coals, plays a particularly important role in the gas diffusion mechanism in the pore network and the amount of gas uptake by the adsorbents. The most often reported critical parameter of the adsorbent sorption capacity is the size of the surface area and the number of active centers located on it. However, due to the heterogeneous structure of coals, the effect of the molecular sieving may play a more significant role in gas adsorption, especially when the size or shape of pores makes the penetration of adsorbates into the inner micropore structure of the adsorbents difficult. Such an effect can be more visible in the case of large size gas molecules, such as propylene (the kinetic diameter over 0.40 nm) and narrow micropores. In the study presented, the effect of a wide range of pores from 0.45 nm to 40 nm on the propylene sorption was tested.

## Materials and methods

### Adsorbents

Coal samples used in this study as adsorbents were obtained from active coal mines located in the Upper Silesia Coal Basin, Poland. The coals were ground in a laboratory mill to reduce the size of the samples and then sieved to range from 0.5 mm to 0.7 mm particle size. Each of the coal samples was stored in amber glass bottles for further adsorption tests. The characteristics of the adsorbents are given in Table 1.

**Table 1:** Physical and chemical parameters of the studied coals.

Parameters		Coal sample no				
		1	2	3	4	5
Ultimate analysis	Carbon, %w/w	63.98	64.37	74.16	86.19	83.60
	Hydrogen, %w/w	4.12	3.68	4.18	4.61	4.84
	Oxygen, %w/w	9.43	9.26	8.19	2.96	2.78
	Nitrogen, %w/w	0.89	0.85	1.23	1.49	1.32
	Sulfur, %w/w	1.57	1.32	0.17	0.48	0.94
Proximate analysis	Ash, %w/w	12.06	12.73	10.54	3.79	6.05
	Moisture, %w/w	8.08	7.84	1.53	0.68	0.54
	Volatile Matter, %w/w	29.2	23.38	25.65	19.27	21.88
	Mineral Matter, %vol	10	10	13	6	12
Petrographic analysis	Vitrinite Reflectance, %	0.55	0.51	0.93	1.41	1.23
	Vitrinite, %vol	55	44	52	67	72
	Liptinite, %vol	14	5	4	0	0
	Inertinite, %vol	31	51	44	33	28

Moisture, ash and volatile matter contents were done by using the determined with the use of automatic thermogravimetric analyzers LECO TGA 701 and MAC 500, while sulfur content was determined by an automatic analyzer TruSpec S by LECO^44^. The procedures for determining moisture and ash were applied according to standards ISO 1,171:2010 and ISO 589:2008, respectively; for volatile matter—ISO 562:2010, whereas for sulfur—ISO 334:1992. Carbon, hydrogen and nitrogen contents were measured with the application of an automatic analyzer TruSpec CHN by LECO in compliance with standards ISO 29,541:2010^45^. Oxygen content was quantified by difference: 100%—moisture—ash—carbon—hydrogen – sulfur. Mineral matter and macerals groups (vitrinite, liptinite and inertinite) according to the method specified in standard ISO 7,404–3:2009.

The characterization of porous structure of coals tested was performed based on the measurements of nitrogen sorption at 77 K and carbon dioxide sorption at 273 K. The analyses were performed with the application of a gas sorption analyzer Autosorb iQ (Quantachrome Instruments, Boynton Beach, FL, USA). The specific surface area was determined with the use ofthe multi-point Brunauer–Emmett–Teller (BET) method^46^ and the nitrogen sorption isotherm data, andthe pore size distribution with the application of the density functional theory (DFT)^47^, while the total pore volume was calculated as the volume relevant for the relative pressure of 0.99. The narrow microporosity of the carbon materials tested in terms of pore area, volume and micropore size distribution was further characterized with the use the carbon dioxide sorption data and the Monte Carlo (MC) method ^48^. The results are given in Table 2.

**Table 2:** Porous structure properties of the studied adsorbents determined with the use of nitrogen sorption isotherm at 77 K and carbon dioxide sorption isotherm at 273 K.

Sample no	Specific surface area, m^2^/g	Total pore volume, cm^3^/g	Average pore diameter, nm	Pore area, m^2^/g	Pore volume, cm^3^/g	Mode diameter, nm
	N_2_ isotherm ^–^196 °C			CO_2_ isotherm 0 °C		
1	41.37	0.054	5.17	213.65	0.080	0.57
2	36.14	0.047	5.24	208.40	0.078	0.63
3	13.97	0.020	5.78	139.03	0.055	0.55
4	7.60	0.012	6.48	115.45	0.050	0.82
5	12.36	0.011	3.67	102.55	0.045	0.82

**Figure 1:**
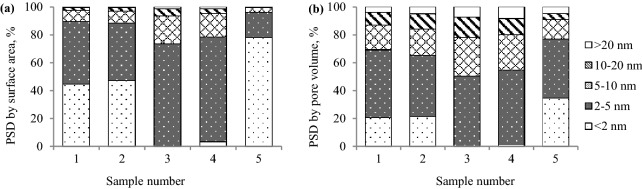
Pore size distribution (PSD) defined by (**a**) pore area and (**b**) pore volume based on the nitrogen isotherm at 77 K and DFT method.

**Figure 2:**
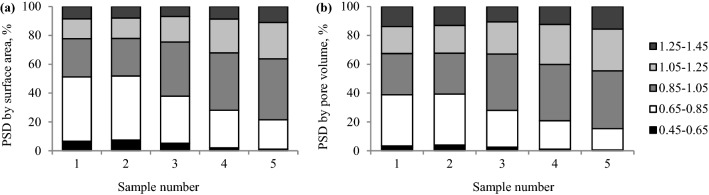
Pore size distribution (PSD) defined by (**a)** pore area and (**b**) pore volume based on the carbon dioxide isotherm at 273 K and Monte Carlo method.

The results of porous structure parameters obtained based on the nitrogen isotherm and the DFT method are given in Fig. 1, while the results calculated based on carbon dioxide isotherm and the Monte Carlo method are shown in Fig. 2.

**Figure 3:**
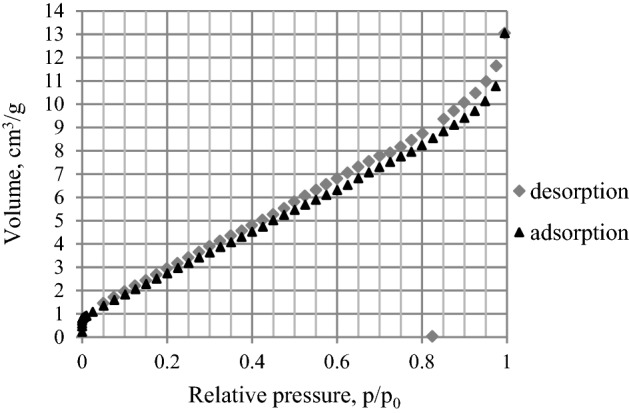
The nitrogen adsorption–desorption isotherm at 77 K for sample 3.

Figure 3 illustrates the exemplary nitrogen isotherm for sample 3 with the hysteresis loop characteristic for mesoporous materials with irregular slit-like shape of pores^49^. The increase in the slope at the final p/p_o_ values is associated with the presence of a certain number of macropores.

### Adsorbates

Three gaseous mixtures used in this study as adsorbates were provided by Air Products Industry Co., Ltd. The compositions of the mixtures are summarized in Table 3.

**Table 3:** Compositions of gaseous mixtures.

No	Gaseous mixture composition
M1	10 ppm C_3_H_6_ (in nitrogen)
M2	5 ppm C_3_H_6_ + 10 ppm C_2_H_4_ (in nitrogen)
M3	10 ppm C_3_H_6_ + 100 ppm C_3_H_8_ + 10 ppm C_2_H_4_ + 100 ppm C_2_H_6_ (in nitrogen)

The composition of the inlet gas stream containing a mixture of ethylene, propane, ethane and propylene reflected the gases content in mixture liberated during the combustion of coal at temperatures above 373 K^11,50^.

### Experimental setup and procedure

In this study, a fixed bed column method has been recognized as an efficient approach for investigating the adsorption process of propylene as one of the fire indicators generated during coal spontaneous combustion. The column method is mainly applied in the reduction of pollutant emissions, through the removal of ions by an ion-exchange bed or the separation of toxic organic compounds by adsorption on carbon materials^51,52^. The fixed-bed adsorption occurs in an open system where the adsorbate continuously passes through a column filled with adsorbent.

**Figure 4:**
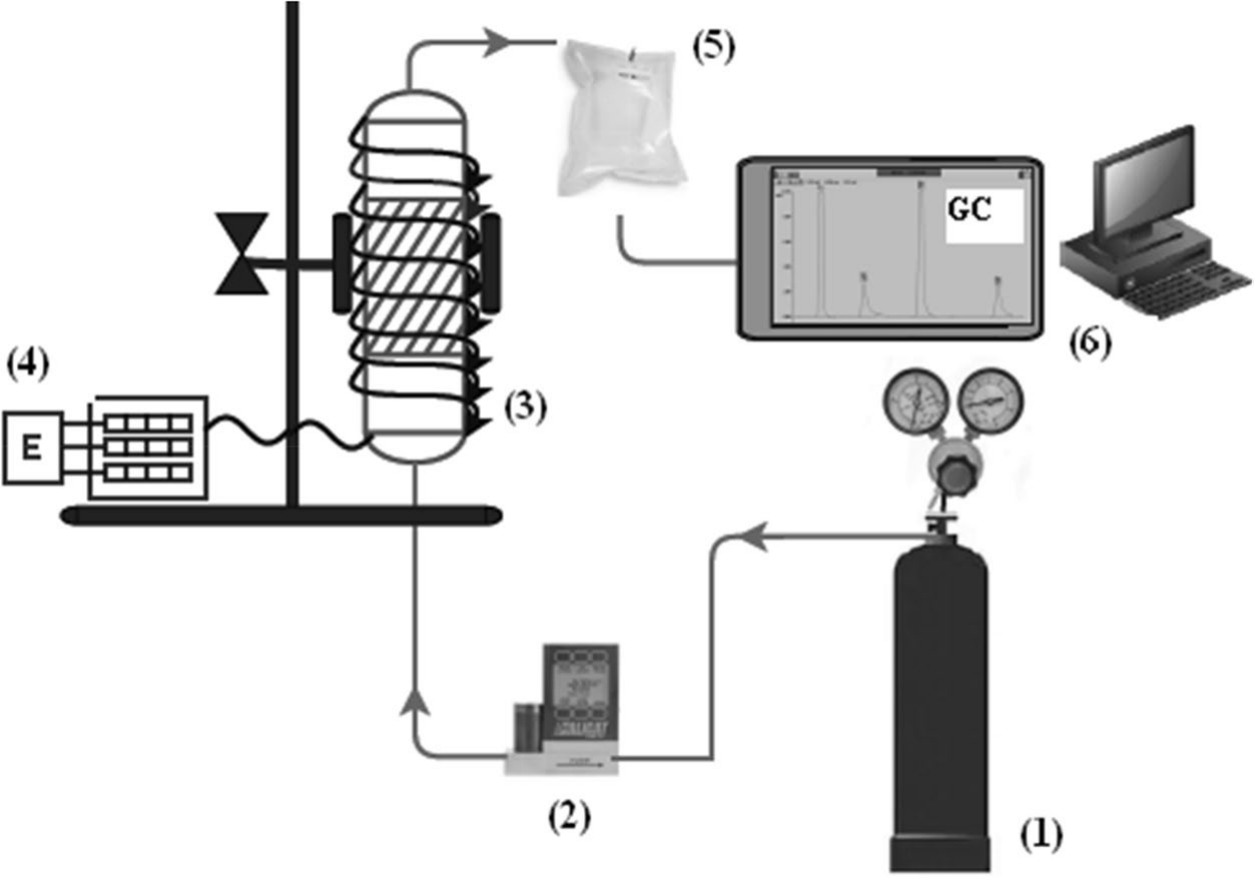
The schematic of experimental set-up for fix-bed adsorption study (1–gas cylinder, 2–gas flow controller, 3–fixed-bed column, 4–control module, 5– sample collection to Tedlar bag, 6–gas chromatography).

The dynamic adsorption tests of propylene on carbon materials were carried out using the laboratory equipment presented in Fig. 4.

The main characteristics of the fixed-bed column and the operating conditions are given in Table 4.

**Table 4:** Operating conditions of the experiment.

Input parameters	Values
Length of column, m	0.60
Diameter of column, m	0.017
Average weight of adsorbent, kg	0.08
Particle size of adsorbent, mm	0.5–0.7
Average density of adsorbent, kg/m^3^	612
Fixed-bed height, m	0.6
Temperature, °C	25
Flow rate, m^3^/s	4.17 × 10^–7^
Time range, min	0–570

The measurement is based on a continuous flow of gaseous mixtures through a single glass column filled with crushed coal at a constant rate of 4.17 × 10^–7^ m^3^/s, calculated on the cross-section of an empty column. The column was kept at the temperature of 298 K by means of a heating cable wrapped around the entire length of the column, and managed by an external controller module. The sorption column was additionally protected by thermal coating to keep the temperature constant during the whole test. The pressure and flow rate at the inlet to the sorption column were controlled with the application of the ALICAT Scientific MS-50 gas flow regulator. The sampling at the outlet of the column was performed at regular time intervals: the first six samples were collected every 5 min, while the remaining samples were collected every 30 min until the end of the test. The adsorption process was stopped after 570 min.

The quantitative analysis of propylene in the mixture was performed by using a Hewlett-Packard-HP 6,890 GC System, Agilent Technologies Inc. gas chromatograph. The chromatograph was equipped with a packed column with alumina ype F1 with helium as a carrier gas and flame ionization detector (FID). This type of detector is sensitive to most hydrocarbons; therefore it is commonly used in industry^53^. The volume of gaseous sample introduced to the chromatograph was of 60 mL. The oven temperature was set at 323 K for five minutes and then the temperature increased at the rate of 308 K/min until it reached 403 K which was held for 2.5 min. The unknown propylene concentration in the mixture was calculated using a calibration curve established by measuring a series of reference gases with various concentrations fitted to the working range. The lowest concentration of propylene that could be reliably measured by this analytical method was 0.01 ppm.

## Results and discussion

**Figure 5:**
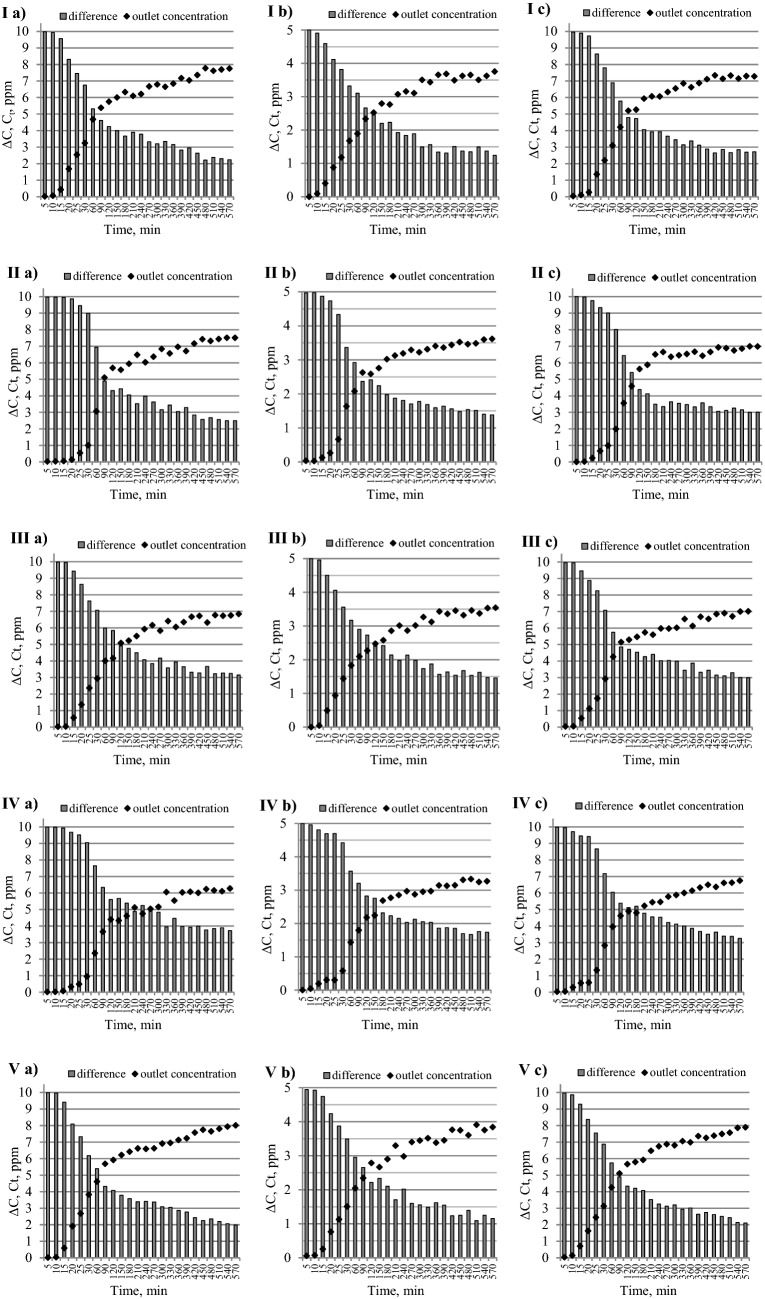
The changes of propylene concentration in the mixture (**a)** M1, (**b)** M2 and (**c)** M3 moving through the sorption column filled with a coal sample: (**I)** 1, (**II)** 2, (**III)** 3, (**IV)** 4 and (**V)** 5.

The effect of using five adsorbents with carbon content ranging from 63.98% to 86.19% on propylene adsorption from three mixtures M1, M2 and M3 is shown in Fig. 5. Column graphs in Fig. 5 illustrate the difference (ΔC = C_o_–C_t_) between the propylene concentration at the inlet gas stream (C_o_) and at the outlet gas stream (C_t_) of the column over a period of time, while the curves show the outlet propylene concentration at any given time.

The results show that the time in which the propylene concentration is equal to, or over 95% of the initial concentration in the measurement time was not reached. In the literature, this time is called saturation time in which a fixed-bed is no longer able to adsorb more gases^48,54^. From the point of view of fire hazard assessment, the saturation time provides information on how long the sorption phenomenon may occur and cause the decrease in the concentration of propylene in the underground atmosphere. As can be seen, at the end of the measurement (at 570 min), the differences ΔC = (C_o_–C_t_) were in the range of 2.03–3.73 ppm (for mixtures M1 and M3) or 1.16–1.74 (for mixture M2), which corresponds to approx. 70–80% of the initial concentration. Under real conditions, the gas flow rate is lower than in the test because of the stresses imposed on the rock layer by the weight of the overlying material. As demonstrated in many works, with the decrease in the gas flow rate, the residence time of adsorbants in the column is prolonged^55,56^. It means that in underground mines, the time of reaching the 70–80% of the initial concentration can be much longer than that which was achieved in the study presented.

As can be observed in Fig. 5, almost all the curves showing the changes of outflow concentration of propylene at the top of the sorption column display a shape of a logarithm function, with the exception of the curves of samples 2 and 4 which constituted the column filling. The shape of the curves for these two samples was similar to a logistic function shape (a typical S-profile). For samples 1, 3 and 5, the time at which C_t_/C_o_ = 0.05 (called breakthrough time) was obtained much faster, within 10–15 min, than for samples 2 and 4^51^. For samples 2 and 4, the breakthrough occurred 20–25 min after the start of the test. It means that propylene was adsorbed more effectively by the lower layers of the fresh samples 2 and 4 during the initial stage of the operations. In contrast, the immediate propylene breakthrough for samples 1, 3, and 5 may suggest that the gas was unable to pass through the narrow pores and to adsorb on the internal surface. After the breakthrough point, the curves showed a sharp increase because of the gradual exhausted of the fixed-bed. The coal mass in the column became less efficient in adsorbing the gas molecules. It was observed that in the time range of 60–90 min, the outlet gas concentration reached 40–50% of the initial concentration; the exception was sample 4 which showed only 20–30% of the initial concentration at this time. After the outlet propylene concentration reached 50–60%, the outflow gas concentrations from column started to rise very slowly, the growth was of the order of 10% or less. The flow of propylene through the pore system became more difficult. This slowdown was probably due to higher diffusion resistance caused by the adsorption process.

**Figure 6:**
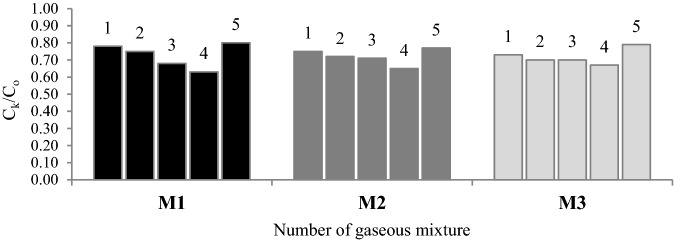
Results for the C_t_/C_o_ ratio for all of the tested coal samples.

Figure 6 shows the ratio of propylene concentration leaving the sorption column at the end of the test (C_k_) to the propylene concentration entering the column at the bottom of column (C_o_).

**Figure 7:**
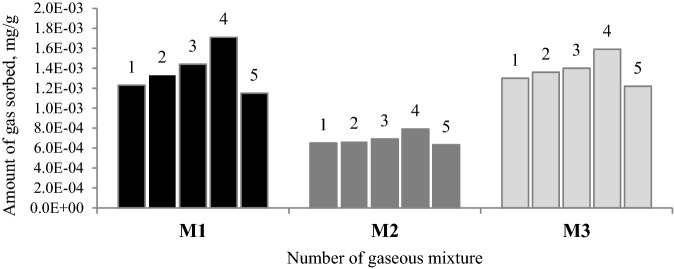
Sorption capacities of tested coals obtained during the laboratory experiment.

In Fig. 7, a maximum sorption capacity achieved by selected coals within the experiment time is presented.

As demonstrated in Figs. 6 and 7, sample 4 was characterized by the lowest values of the ratio in the range of 0.63–0.67 and showed the highest adsorption capacity, while sample 5 had the highest values of the ratio, of the order of 0.77–0.80, and sorbed the lowest amount of gas. Sample 4 has the highest values of the average pore diameter, and the largest share of mesopores of 2–5 nm diameter in pore volume and area (see Table 2 and Fig. 1). Coal sample 5 is not characterized by a porous structure as rich in mesopores as sample 4. In sample 5, the micropores of the pore size of 0.85–1.05 nm determined on the basis of the carbon dioxide adsorption, constituted approx. 76% of the micropores area and 32% of the micropores volume (see Fig. 2). The percentage of mesopores contribution to the porous structure of sample 5 was lower than for the remaining samples and ranged to around 18% in terms of surface area and over 40% when the pore volume is considered. The average pore diameter for sample 5 was the smallest of all in this study and equaled 3.67 nm. The pore size distribution data for narrow micropores based on the carbon dioxide sorption at 273 K (see Fig. 2) for samples 4 and 5 is similar. The difference was reflected in a 10% higher share of pores of 0.85–1.45 nm diameter and a 23–25% lower share of pores of the diameter from 0.45 to 0.85 nm in the volume and area of pores for sample 5 than for sample 4. The results revealed the higher importance of pores with a diameter size over 2 nm over the micropores of the size below 1.45 nm for the propylene uptaken by the coal adsorbents. As shown in Figs. 6 and 7, sample 3 has higher values of the C_k_/C_o_ ratio and lower sorption capacity than sample 4. This coal sample is characterized by lower the average pore size and the share of 2–5 nm pores compared to sample 4. In sample 3, pores of 2–5 nm diameter contribute to about 50% of pore volume (see Fig. 1).

Sample 1 and 2, characterized by comparable values of physical and chemical parameters (see Table 1), had different values of the C_k_/C_o_ ratio and sorbed different amounts of propylene. The values obtained for sample 2 may be attributable to the higher value of inertinite maceral group. The inertinite structure is mostly mesoporous or macroporous^21^. However, in Figs. 1 and 2, it can be observed that samples 1 and 2 show a similar pore size distribution in terms of the area and volume of pore quantified based on nitrogen and carbon dioxide sorption isotherms. The share of pores with a diameter size of 2–5 nm and 1–2 nm in samples 1 and 2 was the highest and amounted to about 40% (by volume) for both ranges. In the above samples, the pores of the 0.65–0.85 nm diameter constituted a larger group (see Fig. 2) which involved approximately 35–36% of the micropore area and 44–45% of the micropore volume. A multimodal pore structure of coal makes that carbon materials may behave as a molecular sieve and block the gases with larger size pass through the micropores and mesoporous channels. The differences between the samples may result from a slightly higher value of the average pore diameter for sample 2. It was noticed that although the surface area of samples 1 and 2 is 2 times (based on carbon dioxide isotherm) and 5 times (based on nitrogen isotherm) larger than that of all the other samples, these samples showed lower propylene sorption capacity (see Table 2) than sample 4.

The various values of the *C*_k_/*C*_o_ ratio as well as various amounts of adsorbed propylene obtained for all the selected samples can be caused by different diffusion rates in the pore structure. In coal characterized by large pore diameters, easier flow of propylene molecules leads to the higher adsorption of the gas. However, in coal characterized by a small pore size, propylene molecules can be trapped in narrow pores due to large-size (0.45 nm) and partially block the access of other propylene molecules to the adsorption sites on the inner surface. Besides, propylene flowing through the porous structure of coal may undergo multilayer adsorption, which may cause the reduction of the pore diameter to smaller sizes^57^. As a result of the multilayer adsorption, the effective pore size may approach the size of a propylene molecule. Based on the results, it can be implied that the relatively high values of pore diameter and the higher share of mesopores in samples 3 and 4 enabled the diffusion of propylene into inner pores and increased the access to more sorption sites on the coal surface. The positive correlation between the pore diameter and sorption capacity was more visible in the case of mixture 1, as can be observed in Fig. 7. Gas transport through porous materials can be controlled by various mechanisms. In pores with sizes larger than the adsorbed gas molecules, the diffusion process will occur mainly in accordance with the Knudsen mechanism in which the molecules collide with the pore walls^58^. In pores with comparable or smaller sizes than the adsorbate diameter, the gas will move both through the collisions of molecules with pore walls, and along the sorbent surface where gas transport is driven by surface energy heterogeneity. Thus, in the case of mesopores the transport of molecules depends on their molecular weight, while in micropores it depends on the size and shape of the diffusing molecule.

The results presented in Figs. 6 and 7, it can be found that the presence of propane, ethane and ethylene in the mixture caused a slight change of the C_k_/C_o_ ratio. The increase in the values of the C_k_/C_o_ ratio for samples 3 and 4 could be due to the fact that in the first stage of the process, smaller particles, i.e. ethylene, are early adsorbed. Larger adsorbed particles need more time to reach the active centers on the inner surface, the number of which has decreased as a result of the earlier adsorption of ethylene. Besides, the ethylene multilayer adsorption will not cause such a significant reduction in the pore diameter as in the case of propylene. A 50% lower concentration of propylene in mixture M2 than in M1 causes that fewer molecules are trapped in coal porous structure resulting in an easier access to more active sites. Interestingly, the share of propane, ethane and ethylene in the mixture led to the rise of propylene adsorption (see Fig. 7) on samples 1 and 2 with the highly developed surface areas. The finding indicates that the presence of other hydrocarbons leads to the increase in the propylene diffusion into the deep inner surface. However, this issue requires further research.

The issue of propylene sorption on coal from a multi-component mixture released during thermal coal oxidation is important for the correct assessment of the degree of endogenous fire risk in underground mines. The porous structure characterization indicated that the e porous structure of coal provides good storage capacity and an ideal transport system for migrating gases. The consequence of such a diversified coal structure is the possibility of migration of gases emitted from the fire center through the coal structure and sorption at adsorption centers. The results of this study showed that the concentration of propylene can be reduced by over 50%. The accumulation of fire gases in the pore structure of coal may result in lowering the value of their concentrations in gaseous samples collected at stations of early detection of mine fires. Numerous studies of the process of self-ignition of coal have shown that with the development of a fire, characteristic gases such as carbon monoxide, carbon dioxide, hydrogen, propylene and ethylene begin to appear in the mine atmosphere^59,60^. The concentration of these gases rises up with the increases in coal temperature. The underestimation of concentration resulting from gas sorption can lead to an incorrect assessment of the development of fire level and a life hazard to employees.

## Conclusion

In this work, all selected coals had a potential to adsorb propylene from the mixtures. A complete gas saturation of the fixed-bed was not reached under experimental conditions due to the slow velocity gas flow. The slowdown of the gas flow was observed after reaching 50–60% of the initial concentration at the exit of the column. It was found that the overall sorption process was controlled through the gas diffusion rate in the pore network. According to the results, the amount of hydrocarbon uptake during the flow through the fixed bed column was correlated with the average pore diameter of coal. This relation was more visible in the mixture of pure propylene and nitrogen in the inlet of column. The highest propylene adsorption capacity values of 1.59∙10^–3^ mg/g and 1.70∙10^–3^ mg/g were obtained for sample 4 with the highest value of the average pore size of 6.48 nm. It was found that the samples of higher share of mesopores and a higher value of the average pore diameter were more efficient in propylene sorption. Even though the higher specific surface area means a large number of active centers and it is generally desired in the selection of the adsorbents, it could be inferred that larger pore sizes are more beneficial for the gas flow into the narrow pores of coals, and, therefore advantageous for propylene adsorption on the carbon materials. It was noticed that the presence of ethylene, ethane and propane in the mixture did not affect significantly the propylene uptake.

The experimental results imply that the concentration of propylene in the mine atmosphere may be lower than the gas concentration in model tests performed in laboratory where the sorption process is not taken into account. In underground conditions. The long retention time and high sorption of propylene on coal may be significant for the detection of endogenous fire on the basis of the concentration of this hydrocarbon. Therefore, the use of propylene as a fire indices reveal justifiable limitations, which has been proven in the course of the experimental works; especially, in coals with a high share of mesopores where easier propylene diffusion will lead to higher gas adsorption on coal surface. The research on the role of pore structure in propylene behavior in porous media can be also helpful during the design of high-performance adsorbents used in the propane/propylene separation.
